# A Case Report of Cutaneous Larva Migrans Treated With Naakupoochi Kudineer, a Traditional Siddha Medicine

**DOI:** 10.7759/cureus.80281

**Published:** 2025-03-09

**Authors:** Saravanasingh Karan Chand Mohan Singh, Nithyamala I, Kaviyarasi Rajaraman, Anbarasan Balasubramanian, Gayatri R

**Affiliations:** 1 Department of Maruthuvam (Siddha General Medicine), National Institute of Siddha, Chennai, IND; 2 Department of Siddha Pharmacology, National Institute of Siddha, Chennai, IND; 3 Department of Noi Naadal, National Institute of Siddha, Chennai, IND

**Keywords:** ancylostoma species, cutaneous larvae migrans, helminthic infection, naakupoochi kudineer, siddha management

## Abstract

Cutaneous Larva Migrans (CLM) is a helminthic infection that is prevalent in tropical and subtropical regions. It is caused by zoonotic hookworms, predominantly the Ancylostoma species, which infrequently infect humans. Hookworms primarily inhabit the small intestine of canines and felines, where adult worms release eggs that are excreted in the host’s feces into the environment. Human infections occur through direct contact with contaminated soil, with humans serving as dead-end hosts for parasites. Clinically, CLM presents as erythematous, serpiginous, and intensely pruritic cutaneous eruptions caused by penetration and migration of larvae. Although the infection is generally confined to the skin, rare instances of larval migration to the lungs can result in more severe complications. A 71-year-old male presented with a progressive, curvilinear, erythematous track in the left upper quadrant region of the abdomen, accompanied by intense pruritus, a burning sensation, and a localized nodular lesion. Erythema was observed approximately 3-4 cm from the site of larval penetration, with the serpiginous tract extending approximately 10 cm in length. The diagnosis of cutaneous larva migrans (CLM) was established based on the characteristic clinical presentation. The Siddha herbal formulation Naakupoochi Kudineer was administered 60 ml twice after meals for seven days, resulting in gradually reducing symptoms and resolving the tract over the following weeks. This study highlights the safety and effectiveness of a Siddha herbal formulation in the treatment of CLM.

## Introduction

The etiological agents of Cutaneous Larva Migrans (CLM) include hookworms, such as *Ancylostoma caninum*, *A. braziliense*, *A. duodenale*, and *Necator americanus*. These parasites excrete eggs in the faeces of infected canines and felines, which hatch within 24 hours and develop into infected larvae that persist in the soil. Upon contact with human skin, larvae penetrate and migrate in a characteristic serpentine pattern [1]. Lesions typically manifest as erythematous papules that progressively expand at 1-2 cm/day. Vesicular or follicular lesions may also develop [2]. Addressing CLM as a significant public health issue is crucial for effectively combating this disease in developing countries such as India. Prevalence reached 8.2% in population-based studies, with considerable variation between sexes and age groups.

CLM is an erythematous, serpiginous, pruritic cutaneous eruption caused by accidental percutaneous penetration and subsequent migration of the larvae [3]. CLM is typically distributed on the lower distal extremities, including the dorsum of the feet, and interdigital spaces of the toes, anogenital region, and buttocks, and less commonly affects the dorsal surface of the anterior abdominal wall, penile shaft, perianal area, sole, and oral cavity. *Ancyclostoma braziliense *is the most common causative agent in humans. In CLM, the parasite's life cycle begins as these hookworms generally live in the intestines of domestic pets, such as dogs and cats. Their eggs pass from animal faeces into warm, moist, sandy soil, where the larvae hatch under favourable conditions to infective larvae that penetrate the host epidermis but cannot penetrate the dermis layer of the skin [4]. Following penetration, larvae remain in the stratum germinativum. The active production and secretion of hyaluronidase seem to render the movement of the larvae possible and advance at a rate of 2-3 cm per day [5]. CLM initially manifests as local pruritic linear erythematous papules. Later, these classic clinical features may be superimposed with impetigo following secondary bacterial infections from repeated scratching.

As the larvae survive in the human host for 2-4 weeks, the disease is self-limited in over 80% of cases. It is now treated with anthelmintic agents, such as albendazole and ivermectin. Although albendazole is used to treat CLM, it has side effects such as fatal pancytopenia [6]. The primary mechanism of action is inhibition of microtubule polymerization binding to β-tubulin, like colchicine, as a microtubule formation inhibitor. It is reasonable that albendazole can cause side effects such as alopecia and cytopenia [7]. Albendazole can cause bone marrow suppression, leading to pancytopenia, as evidenced by several case reports. The drug's metabolite, albendazole sulfoxide, is implicated in this adverse effect, particularly when its serum concentration is elevated due to prolonged use or overdose [8]. In animal studies, albendazole administration has been linked to bone marrow hypoplasia, indicating acute injury to the bone marrow cells [9].

These side effects are rare but potentially serious events, whereas Siddha, a traditional system of medicine, effectively and successfully treats CLM with a combination of herbal medicines. Previous studies have demonstrated regression of the disease [10]. This study aimed to evaluate Siddha medicine's efficacy in curating CLM.

## Case presentation

Background

A 71-year-old male patient residing in the coastal region of Chennai presented with complaints of a creeping, serpiginous, erythematous tract accompanied by intense pruritus and a burning sensation in the left upper quadrant of the abdomen for one month. His travel and family history revealed no significant findings. He had not sought any prior medical intervention for his condition.

Clinical examination

Upon physical examination, an approximately 11-cm curvilinear, erythematous, serpiginous tract was observed, accompanied by a localized lesion with a distinctive nodular tip over the left upper quadrant of the abdomen. Eosinophilia was absent in the complete blood count analysis. The peripheral smear appeared normal, and no abnormalities were detected in the chest X-ray. It shows that it had not caused any systemic complications. No similar lesions were identified elsewhere on the body.

Siddha treatment

**Figure 1:**
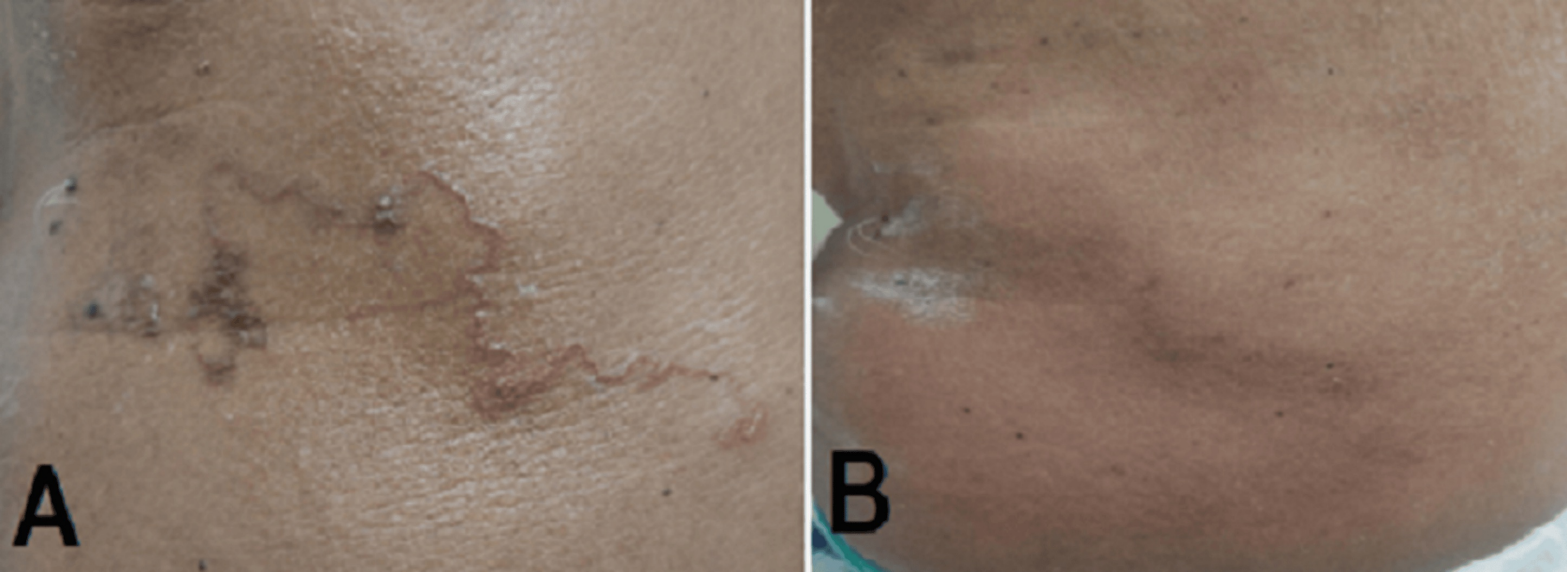
Progress of skin lesions in the abdomen before and after treatment. A. Erythematous, serpiginous lesions of cutaneous larva migrans on the left upper quadrant of the abdomen; B. Complete resolution one week after treatment, with only the presence of post-inflammatory hyperpigmentation.

The medicine was administered with the prerequisite of written consent. Naakupoochi Kudineer, a traditional herbal formulation, was prescribed to the patient at a dose of 60 ml twice daily, postprandially, for one week. The patient showed good compliance with the prescribed treatment regimen and consumed the herbal decoction for one week. Interestingly, a gradual improvement in the symptoms associated with cutaneous larva migrans (CLM) was observed during this period. The patient reported a progressive reduction in pruritus and burning sensation, characteristic features of CLM. Furthermore, the erythematous serpiginous tract, a pathognomonic sign of CLM, exhibited significant resolution over the subsequent weeks following the completion of the Naakupoochi Kudineer treatment. The remarkable alleviation of symptoms and resolution of the serpiginous tract highlight the potential of Naakupoochi Kudineer as an effective therapeutic option for the management of CLM. The progress of skin lesions in the abdomen before and after treatment is shown in Figure 1.

## Discussion

Naakupoochi Kudineer is a fascinating polyherbal preparation made up of eight medicinal herbs, each carefully proportioned to maximize their benefits. The ingredients include *Butea monosperma *(13.33%), *Nigella sativa *(13.33%), *Embelia ribes *(13.33%), *Tachyspermum ammi *(13.33%), *Operculina turpethum *(13.33%), *Cassia angustifolia *(13.33%), *Foeniculum vulgare *(13.33%), and *Picrorhiza kurroa *(6.66%) [11]. Each of these herbs brings unique pharmacological properties that work together to enhance the therapeutic efficacy of Naakupoochi Kudineer. This traditional Siddha herbal formulation is especially known for its antihelmintic and larvicidal properties, effectively serving as a dewormer against CLM nematodes. For instance, the seeds of *Butea monosperma *are rich in anthelmintic compounds like butin, butein, and benzothiazole, which have proven effective in fighting parasitic infections [12]. Similarly, *Nigella sativa *is packed with bioactive constituents such as thymoquinone, carvacrol, and alpha-hederin that contribute to its anthelmintic effects [13]. *Embelia ribes*, on the other hand, has showcased a broad range of biological activities, particularly its significant anthelmintic effects primarily due to embelin, effective against various parasitic infections [14]. The phytochemicals found in *Tachyspermum ammi*, including thymol, saponins, and glycosides, further enhance its anthelmintic capabilities, with thymol noted for its nematicidal properties [15, 16]. *Foeniculum vulgare *seed extract has also shown promise, demonstrating effectiveness against *Gastrothylax crumenifer*, which underscores its potential role in treating helminthiasis in livestock [17]. Meanwhile, the rhizomes of *Picrorhiza kurroa *contain iridoid glycosides, like picroside I and II, known for disrupting the physiological functions of helminths, ultimately leading to their expulsion from the host [18].

Conventional anthelmintic treatments like albendazole and ivermectin are generally effective and well-tolerated, although they may come with side effects such as liver enzyme abnormalities, hypotension, bone marrow suppression and central nervous system effects. In contrast, cryosurgery options, including liquid nitrogen or ethyl chloride spray, are no longer recommended. Studies suggest that cryotherapy is less effective than anthelminthic treatments, often failing to eliminate larvae and leading to persistent symptoms, while also posing risks of skin damage and other side effects [19, 20].

In the traditional Siddha system of medicine, herbal formulations like Naakupoochi Kudineer are widely used as dewormers to combat CLM. The synergistic action of its various herbal components contributes to its anti-helminthic, anti-inflammatory, and wound-healing properties. Nevertheless, further research is essential to unravel the exact mechanisms that underpin the therapeutic effects of Naakupoochi Kudineer in treating CLM.

Mode of action

The synergistic action of the various herbal components in Naakupoochi Kudineer significantly contributes to its anti-helminthic properties. These herbal constituents may exert their effects by binding to the beta-tubulin of nematodes, thereby inhibiting the process of microtubule polymerization. This inhibition substantially affects cellular processes, disrupting cell division and energy metabolism within the parasites, ultimately leading to their death.

Case benefits

The treatment outcomes were encouraging, with the patient showing a progressive decrease in symptomatology. Over the subsequent weeks, the serpiginous tract resolved completely. At the three-month post-treatment follow-up assessment, a substantial amelioration was noted, with no drug-related adverse events or relapse observed.

Limitation of the study

This study is limited by its nature as a single case report, which necessitates further systematic clinical studies involving larger populations to validate the effectiveness of the remedy used. While the case provides valuable insights, it does not offer robust evidence due to its susceptibility to biases and heuristics that can influence clinical practice and permeate scientific literature. Additionally, this type of study does not generate epidemiological data, making it challenging to draw generalizations or comprehensive inferences for managing CLM in broader contexts. Consequently, no definitive conclusions can be made based solely on a single case report regarding the treatment and management of this condition in future cases.

## Conclusions

Practitioners should consider the potential for Hookworm-induced Cutaneous Larva Migrans (CLM) in patients with a history of travel to tropical regions, mainly if they have engaged in barefoot walking. This research underscores the potential of plant-based therapeutics in managing primary dermatological conditions. It can be concluded that Siddha medicine Naakupoochi Kudineer effectively treats CLM. Furthermore, the findings emphasize the importance of Siddha medicine in disease treatment and underscore its unique and significant role in healthcare. These investigations validate the efficacy and relevance of Siddha medicine in contemporary healthcare while shedding light on the rich botanical resources that form the basis of this ancient healing tradition. It is necessary to do additional research on a larger sample to establish that Siddha medications provide an effective method for managing CLM.
